# Primary care clinicians’ attitudes towards point-of-care blood testing: a systematic review of qualitative studies

**DOI:** 10.1186/1471-2296-14-117

**Published:** 2013-08-14

**Authors:** Caroline HD Jones, Jeremy Howick, Nia W Roberts, Christopher P Price, Carl Heneghan, Annette Plüddemann, Matthew Thompson

**Affiliations:** 1 Department of Primary Care Health Sciences, University of Oxford, Woodstock Road, Oxford, UK; 2 Bodleian Health Care Libraries, Knowledge Centre, University of Oxford, Headington, Oxford, UK

**Keywords:** Primary health care, Point of care technology, Diagnosis, Qualitative research, Systematic review

## Abstract

**Background:**

Point-of-care blood tests are becoming increasingly available and could replace current venipuncture and laboratory testing for many commonly used tests. However, at present very few have been implemented in most primary care settings. Understanding the attitudes of primary care clinicians towards these tests may help to identify the barriers and facilitators to their wider adoption. We aimed to systematically review qualitative studies of primary care clinicians’ attitudes to point-of-care blood tests.

**Methods:**

We systematically searched Medline, Embase, ISI Web of Knowledge, PsycINFO and CINAHL for qualitative studies of primary care clinicians’ attitudes towards point-of-care blood tests in high income countries. We conducted a thematic synthesis of included studies.

**Results:**

Our search identified seven studies, including around two hundred participants from Europe and Australia. The synthesis generated three main themes: the impact of point-of-care testing on decision-making, diagnosis and treatment; impact on clinical practice more broadly; and impact on patient-clinician relationships and perceived patient experience. Primary care clinicians believed point-of-care testing improved diagnostic certainty, targeting of treatment, self-management of chronic conditions, and clinician-patient communication and relationships. There were concerns about test accuracy, over-reliance on tests, undermining of clinical skills, cost, and limited usefulness.

**Conclusions:**

We identified several perceived benefits and barriers regarding point-of-care tests in primary care. These imply that if point-of-care tests are to become more widely adopted, primary care clinicians require evidence of their accuracy, rigorous testing of the impact of introduction on patient pathways and clinical practice, and consideration of test funding.

## Background

Point-of-care tests (POCTs, also known as near-patient tests) have been used for many years, for example blood glucose and urine testing; yet recently there has been an explosion in the development of these technologies [1,2]. POCTs are usually delivered during a clinical visit; the results are available quickly without the need to send samples to a laboratory. In primary care this could offer alternatives to traditional laboratory testing, with the potential to maintain or improve patient convenience, satisfaction and health outcomes whilst saving time and costs [3-7]. However there are also potential disadvantages including time needed to use them, inaccurate tests, and inappropriate testing [5,8].

Despite their availability and potential to improve patient care, POCTs have not been widely adopted in primary care in most countries. Exploring why this is the case requires (among other things) a clearer understanding of the attitudes of primary care clinicians to the use of these tests, including any concerns they may have. We aimed to gain an understanding of primary care clinicians’ attitudes towards POCTs by conducting a systematic review. This paper reports the results of that systematic review and synthesis of qualitative studies.

## Methods

### Literature search and study selection

We conducted a systematic search for primary qualitative research articles exploring attitudes of any type of primary care clinicians towards POCTs. We defined POCTs as any test where the results are available during a clinical visit, without the need to send sample to a laboratory. We included only studies on POCTs involving blood (excluding other types of sample such as urine or throat swabs) because these could replace venipuncture and laboratory testing, and have the biggest potential to change practice; and attitudes towards them may differ to attitudes towards other types of POCT. We limited our search to primary care and high income country settings (defined by the Organisation for Economic Cooperation and Development) – excluding studies in emergency department, hospital or low or middle income country settings – since attitudes may be specific to different contexts. We included only studies which used qualitative data and analyses, because qualitative data enables in-depth understanding of the range of attitudes of participants from their perspectives. Studies where qualitative data and analyses were embedded within larger mixed methods studies were included only if we could extract the qualitative data. Where studies reported attitudes of secondary care clinicians or patients in addition to primary care clinicians, we extracted and synthesized data relating to primary care clinicians only.

We searched Medline (1948-present), Embase (1974-present), ISI Web of Knowledge (1945-present), PsycINFO (1967-present) and CINAHL (1980-present) from database start date to February 2013. The search was developed through an iterative process combining search terms that best describe our search criteria, including free-text terms and subject headings to reflect the following concepts: ‘point of care test’ and ‘primary care’. Validated search filters were adapted to focus the search to qualitative research papers (for example Wong et al. for Medline [9]). In addition we scanned reference lists of included studies. The search strategy included a search for previously conducted reviews in order to identify primary studies from any relevant previous reviews [10] (see Additional file 1 A for Medline search strategy).

Two authors (CJ, JH) independently assessed the studies for eligibility. Titles were independently screened by both authors and those that were obviously not relevant were excluded. Both authors independently examined the full text of all remaining studies, and those that did not meet the inclusion criteria were excluded. Discrepancies regarding inclusion were resolved by discussion with a third reviewer (MT) until agreement was reached.

### Data synthesis

Systematic review of qualitative evidence has become popular in recent years [11]. There are a number of methods for synthesizing findings from qualitative studies [12]: we conducted thematic synthesis, which is able to integrate the findings of multiple qualitative studies and generate new concepts and hypotheses [13,14].

Two authors (CJ, JH) independently extracted data on study characteristics and context from included studies using a standardized data extraction sheet. We also independently extracted the study results/findings, including the primary data reported in studies (participant responses) as well as authors’ summaries and interpretations. After familiarizing ourselves with the findings of individual studies we developed codes, and assigned sections of findings to these codes. Codes were then grouped into descriptive themes. Codes and themes were not determined a priori but were driven by the data. From the descriptive themes we generated analytical themes reflecting new insights going beyond the findings of the included studies [14]. Themes were discussed amongst all authors and consensus reached. We reflected critically on how our own backgrounds and assumptions impacted our interpretation of the data, and compared the results of our synthesis with the conclusions of individual studies to verify our findings.

The same authors (CJ, JH) appraised study quality using a checklist based on the criteria described by Kuper and colleagues [15]. The checklist included items on whether the sample, data and analysis were appropriate; the results can be transferred across settings; ethical issues were adequately addressed; and it is clear what the researchers did. We took account of study quality when interpreting the findings, by being more cautious when interpreting the results of low quality studies and highlighting possible limitations.

## Results

### Search results

**Figure 1:**
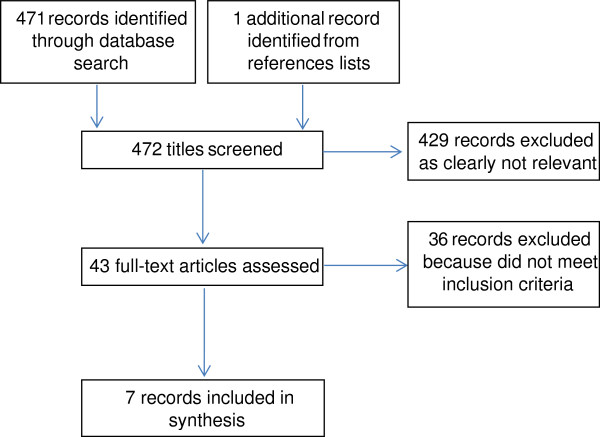
Flowchart of literature search.

The search revealed 472 potentially eligible studies. After title screening, 429 were excluded, and a further 36 were excluded after assessing the full text (see Figure 1).

### Characteristics of included studies

We included seven studies [16-22] with a total of around 200 participants (see Table 1). They took place in Europe (*n* = 6) and Australia (*n* = 1). One was published in 1997, and the remainder were published between 2007 and 2011. All used semi-structured interviews to gather data; two also used focus groups. The 1997 study referred to blood POCTs using samples obtained by venipuncture and analysed onsite in the health centre [19]; the others referred to finger-prick blood tests. All examined attitudes of general practitioners (GPs); two also examined attitudes of nurses.

**Table 1:** Characteristics of included studies

**Primary author (year)**	**Country**	**Type of study**	**Type of POCT**	**Participants’ experience of using POCTs**	**Number of participants**	**Type of primary care clinicians**
Butler (2008) [16]	Wales (United Kingdom)	Semi-structured qualitative interviews	A test to distinguish bacterial from viral infections using a finger-prick blood test	No experience – participants discussed their perspectives on possible introduction of the POCT	40	GPs
Cals (2010) [17]	The Netherlands	Semi-structured qualitative interviews	C-reactive protein POCT for lower respiratory tract infection and other common infections	All participants had been using the POCT for nearly 3 years at the time of interview as part of a randomized trial	20	GPs
Cals (2009a) [18]	The Netherlands	Semi-structured qualitative interviews	C-reactive protein POCT to differentiate serious from self-limiting lower respiratory tract infection	10 participants had used the POCT for at least two years at the time of interview as part of a randomized trial; 10 participants had no experience	20	GPs
Gillam (1997) [19]	United Kingdom	Semi-structured interviews and a focus group	A range of POCTs including haematology (full blood count, platelets); chemical pathology (sodium, potassium, urea, creatine); glucose, cholesterol; bilirubin, alkaline phosphatase, aspartate transaminase; creatine kinase	Participants worked in a health centre where POCTs were piloted; a nurse took blood samples using venipuncture, they were analysed onsite, and the results were made available to the GP at the end of surgery or immediately if requested	Unknown	GPs
Glover (2008) [20]	Australia	Group discussions + individual interviews	INR (international normalised ratio) fingerstick test for monitoring patients on warfarin	No experience (this is not stated explicitly but is assumed)	33 participants in total; unknown how many were GPs and nurses	Hospital pharmacists, specialists, nurses, GPs. We included only the attitudes of GPs and nurses in the review (nurses treated patients in their homes as well as in hospital)
Stone (2007) [21]	United Kingdom	Semi-structured qualitative interviews	HbA1c (glycated haemoglobin) finger-prick test for patients with type 2 diabetes	Participants took part in a pragmatic, open, randomized controlled trial, where they gave some patients usual care and others POCTs for 1 year	11	GPs, practice nurses
Wood (2011) [22]	Belgium, Hungary, Spain, Wales, Poland, Italy, England, Norway, The Netherlands	Semi-structured qualitative interviews	C-reactive protein POCT to aid management of acute cough/lower respiratory tract infection	Participants from Norway routinely used the POCT; participants from other countries had no experience	80	Primary care clinicians

Two studies used data obtained from interviews with GPs participating in the same randomised trial [17,18]. Each had 20 participants, 10 of whom overlapped between the two studies. Since both had 10 different participants, and the focus of analyses were different, we included both in our synthesis.

The type of test included in each study is shown in Table 1. Four studies examined attitudes towards C-reactive protein (CRP) POCTs or hypothetical tests which could similarly distinguish between viral and bacterial infections [16-18,22]: we refer to these as diagnostic. Two examined POCTs for monitoring chronic illness (patients with diabetes [21] and those taking warfarin [20]): we refer to these as monitoring. One examined attitudes towards a range of POCTs [19]. We looked for similarities and differences in attitudes towards diagnostic and monitoring POCTs.

Studies varied according to whether participants had experience using POCTs, were being asked about a test of which they had no experience, or contained a combination of those with and without experience (Table 1). Three studies including participants with experience were conducted in the context of a randomised trial in which a test was introduced as an intervention to all [17,21] or some [18] participants, and one included GPs from a health centre where POCTs were being piloted [19]. Another included GPs from Norway, where CRP POCTs are routinely used, and from eight other European countries where they are not [22]. We looked at similarities and differences in attitudes between clinicians with different levels of experience.

Five of the included studies were of good quality (see Table 2) [16-18,21,22]. Study samples, data collection and analyses were appropriate and they were clearly described. Another study lacked some details about the sample (for example how many of the participants were GPs and nurses, and whether they had any experience at all of using POCTs), and the sample size was small (only one focus group for each group of clinicians); but the methods of data collection and analysis were appropriate [20]. We considered these studies to be relatively equally rigorous and trustworthy and treated them equally in the synthesis. One other study was poorly described [19]: it lacked details including the number of participants; the design, duration and timing of the interviews and focus group; how the data were analysed and by whom; and whether the results include verbatim quotes or not. Therefore it is not possible to assess how rigorous and trustworthy the study is. Therefore, this study did not contribute as much to the synthesis. Additionally of note when interpreting the results of this study, the nature of the POCT in this study differed from the others in our synthesis because it used blood obtained by venipuncture rather than finger-prick blood samples.

**Table 2:** Quality appraisal of included studies

	**Was the sample used in the study appropriate to its research question?**	**Were the data collected appropriately?**	**Were the data analysed appropriately?**	**Can I transfer the results of this study to other settings?**	**Does the study adequately address potential ethical issues, including reflexivity?**	**Overall: is what the researchers did clear?**
Butler [16]	Yes	Yes	Yes	Yes	No	Yes
Cals [17]	Yes	Yes	Yes	Yes	No	Yes
Cals [18]	Yes	Yes	Yes	Yes	No	Yes
Gillam [19]	Unclear	Unclear	Unclear	Unclear	No	No
Glover [20]	Unclear	Yes	Yes	Unclear	No	Yes
Stone [21]	Yes	Yes	Yes	Yes	No	Yes
Wood [22]	Yes	Yes	Yes	Yes	No	Yes

Amongst all studies there was a lack of discussion about the impact of researchers’ characteristics and perspectives, and their relationships with participants. This absence of reflexivity limits our ability to assess the influence of the researchers on the data and interpretations.

Through the synthesis we identified descriptive themes regarding positive and negative attitudes of primary care clinicians towards blood POCTs. From these, we developed three analytical themes which are discussed below. Within each of these themes there are issues which may act as facilitators and barriers to widespread adoption of POCTs in primary care: Table 3 summarises the barriers and facilitators to POCT use within each theme.

1) Impact of POCTs on decision-making, diagnosis and treatment

**Table 3:** Summary of how primary care clinicians’ attitudes towards blood POCTs may act as facilitators and barriers to their adoption in primary care

**Theme**	**Facilitators to adoption of POCTs in primary care**	**Barriers to adoption of POCTs in primary care**
Impact of POCTs on decision-making, diagnosis and treatment	Increased diagnostic certainty	Concerns about accuracy
	More effective targeting of treatment (e.g. antibiotics)	Might not be helpful or alter consultations
		Possible misleading results
Impact of POCTs on clinical practice more broadly	Fewer re-consultations / phone calls for the same or future episodes of illness	Over-reliance, undermining of clinical expertise
	Enhanced confidence and job satisfaction	Cost, equipment maintenance, time
	Avoidance of missing or delayed results, and loss of patients to follow-up	Usefulness limited to certain situations and patients
Impact of POCTs on patient-clinician relationship and perceived patient experience	Enhanced communication through discussing immediate results	Possible patient dislike of testing
	Increased patient education and self-management of chronic conditions	Patient anxiety resulting from intermediate results
	Shared decisions with patients (e.g. antibiotic prescription)	
	Greater reassurance and satisfaction for patients	
	Patient confidence in clinicians’ decisions	

Many attitudes were related to how POCTs might enhance immediate diagnosis and treatment. Diagnostic POCTs were viewed as helpful for improving diagnostic certainty and confidence in clinical decisions; [16-18,22] particularly for ruling out serious infections [17].

POCTs were perceived to enable more effective targeting of treatment. Particularly, tests which could distinguish viral from bacterial infections were considered helpful and could aid decision-making regarding antibiotic prescription: [16-18,22]. *“It also helps you to be a bit more careful in prescribing antibiotics, that’s true. It makes you more aware that you may be using them too often”* (GP [17]). This was a belief of GPs who had both used [17,22] and not used diagnostic POCTs [16,18,22].

A primary concern was the analytical accuracy of POCTs [16,19,20,22]: *“the results they give are not accurate enough”* (Primary Care Clinician [22]), which might lead clinicians to miss serious infections [22]. Clinicians did not feel ‘convinced’ or confident about their performance [16,20]: *“we’ve had no research presented to us”* (Nurse [20]). In one study GPs raised concerns that they would be liable medico-legally for any problems arising from inaccurate results [19] (note this study was poorly described and it is not possible to assess its rigour).

Although POCTs were perceived on the whole to enhance patient care (if tests were accurate), exceptions were noted. A small number of individuals believed that it was not important or always helpful to distinguish bacterial from viral infections, [16] that monitoring POCTs did not influence the outcome of a consultation, [21] or questioned the added diagnostic value [22]. Diagnostic POCTs would not be helpful when serious complications arise from viral illnesses [16]; and misleading results due to CRP not being raised in the early stages of illness, or due to false results, could lead to inappropriate diagnosis and treatment: *“I see the disadvantage that a mistake or false results can come out as a result. So for instance there is a positive result…. But a different and hidden problem can be the cause”* (Primary Care Clinician [22]). Usefulness of monitoring POCTs performed by nurses varied *“according to the nurse’s level of responsibility for making management changes and the availability of a doctor during nurse-led clinics”* (authors [21]). GPs in one study actually felt waiting for results from laboratory testing was advantageous because it gave them time to *“defer decision-making while awaiting results, thereby ‘allowing nature to take its course’”* (authors [19]) (note that this study was poorly described and trustworthiness of findings cannot be assessed).

2) Impact of POCTs on clinical practice more broadly

Further to the direct impact on diagnosis and treatment, POCTs were thought to have a wider-reaching impact. The immediacy of diagnostic POCT results could reduce re-consultations or phone calls regarding the same episode of acute illness [17,22]. Some GPs believed that consultations for future illnesses may also be reduced: *“If you don’t treat a patient with antibiotics [after CRP testing] and the complaints resolve spontaneously, I think that patients will tend to wait and see and not consult the doctor again for the next similar illness episode. So what we hope is that this management including CRP will lead to fewer consultations or repeat consultations for new infections”* (GP [17]). Advantages of monitoring POCTs in terms of future consultations were *“avoidance of missing or delayed results and occasional loss of patients to follow-up”* (authors [21]).

Immediacy of results could enhance clinicians’ confidence and job satisfaction when using monitoring POCTs: *“My confidence has actually grown in discussing the result with them… I feel it’s sort of added and rounded off the consultation”* (Nurse [21]).

There were some concerns that clinical practice could be negatively affected. Clinicians worried about potential over-reliance on diagnostic POCTs, [17,22] undermining of clinical expertise, and over-testing: *“Perhaps it’s being used a bit too often. I think you need to be careful about that”* (GP [17]); *“The disadvantage is that doctors may rely more on test results than on clinical judgement”* (Primary Care Clinician [22]); *“There’s a risk that you let the test determine your management. In the end, what matters is the person who’s sitting there and what you hear and what you find on physical examination”* (GP [17]).

Clinicians also expressed concerns that POCTs could only be used intermittently and in certain situations and patients [16,18,20]: *“for example, in situations where they were unsure of the aetiological cause on the basis of the clinical presentation, or in a situation of deadlock with a patient who definitely wanted antibiotics”* (authors [16]).

Concerns regarding feasibility included cost, [16,19,21,22] maintenance of equipment, [16] quality control, [19] time [16,17,22] and organisational issues (for example interference in nurse activities) [17]. More positively, POCT devices were described as user-friendly, [17,21] and in some cases as having *“very little influence on their [GPs’] workload”* (authors [17]).

3) Impact of POCTs on the patient-clinician relationship and perceived patient experience

Participants felt that being able to discuss results of monitoring POCTs with patients immediately was beneficial for patient-clinician communication, and determining the most appropriate treatment plan [21]: *“you can instigate changes in treatment there and then and discuss it with the patient”* (Nurse [21]). POCTs could therefore enhance patient education and self-management of chronic conditions [20,21]: *“It’d be great for patient advocacy and empowering them to take some responsibility for their own health care”* (Nurse [20]).

Regarding diagnostic POCTs, it was believed that patients would be convinced, reassured and more satisfied in their GP’s decisions if POCTs had been used, compared to if they had received no test [16-18,22]: *“then you can justify what you are saying to the patient. Because nowadays, patients want the evidence as well”* (GP [16]). In particular, a test result confirming a GP’s decision not to prescribe antibiotics would help them to “*sell*” this decision to patients [16] and manage patient expectations for antibiotics, [22] leading to shared decisions with patients [18]. This was perceived by GPs to help preserve a trusting doctor-patient relationship [17]. GPs with different levels of experience of using diagnostic POCTs had similar perceptions that they would help to reassure patients and lead to more effective targeted treatment without alienating or upsetting patients [16,18,22]. GPs in one described that the POCT service *“boosted the practice’s image”*[19] (note that this study is poorly described and rigour cannot be assessed).

Although it was widely believed that patients would like to have POCTs available, concerns that patients may not like testing were mentioned by a minority of participants, [16,17,22] with children mentioned in particular [16]. Furthermore, some GPs were worried about difficulty interpreting and explaining diagnostic test results, [22] particularly intermediate results [17] which could increase uncertainty in patients: *“the patient may think that their blood was not entirely OK, so that may make them insecure and worried”* (GP [17]). With regards to interpreting test results, *“a solid training session was highly valued”* (authors [17]).

## Discussion

### Main findings

Despite considerable heterogeneity regarding the specific tests involved and their purpose (primarily diagnosis or monitoring), we found commonalities in primary care clinicians’ attitudes towards POCTs. Overall, these tests were believed to increase diagnostic certainty, help target treatment, educate and empower patients, and improve the relationship between clinicians and patients by enhancing communication and shared decision-making. A major concern was the need for accurate tests. Clinicians were also concerned about cost, over-reliance – in that POCTs could undermine clinical skills – and limited usefulness. Table 3 summarizes these issues and highlights how they may act as facilitators and barriers to widespread adoption of POCTs in primary care.

The number of included studies was small, and there was heterogeneity regarding the type of test, its purpose, the type of primary care clinicians participating, and whether or not they had practical experience of using POCTs. This may limit generalizability of our results to other tests and settings. However, we identified commonalities; and clinicians with experience of using POCTs routinely (Norwegian GPs [20]) described similar benefits and concerns to those who had experience of using them only in the context of a trial, and those who had not used them. We have highlighted which attitudes were specific to only diagnostic or monitoring POCTs; for example more effective targeting of antibiotics with diagnostic POCTs, and enhanced patient self-management with monitoring POCTs. Each of the three main themes applied to both diagnostic and monitoring POCTs.

Some of these issues are not unique to POCTs, but also apply to laboratory testing in general; for example concerns that misleading results could lead to inappropriate diagnosis and treatment, and that testing is only useful in certain circumstances. However, the majority apply to POCTs specifically due to speed of obtaining (and having to interpret) test results having an immediate impact, for example by improving diagnostic decision-making and communication with patients.

Despite the wealth of POCTs available there has not been widespread adoption of POCTs in primary care to date, at least in most countries. In addition to the barriers to POCT use identified here, other reasons for lack of widespread use may include lack of needs assessments of primary healthcare clinicians, resulting in discordance between the tests that they want/would use frequently, and those that are produced. Furthermore, multiple steps are needed between the development of new diagnostic tests and their adoption into clinical practice: tests can reach the market with limited evaluation of clinical utility or cost-effectiveness in primary care populations [23]. For example, evaluation of readily available POCTs has been inconclusive regarding cost-effectiveness compared to standard laboratory testing, [24,25] and uncertain regarding their clear role in primary care [24].

### Comparison with existing literature

A quantitative study of patient satisfaction with POCTs in general practice similarly highlighted strengthened relationships between patients and GPs, and motivation of patients to better manage chronic conditions [26]. GPs in another survey study supported the use of POCTs in general practice due to improved convenience, patient care and efficiency [27]. Also in keeping with our findings, Hobbs [28] concluded that there are “clinical niches” where POCTs are most likely to influence practice, and that these must be found in order that their potential is realised; and GPs’ use of CRP POCTs was found to significantly reduce antibiotic prescribing [6].

### Strengths and limitations

This is the first systematic review, to our knowledge, to explore primary care clinicians’ attitudes towards a rapidly growing technological area in primary care, namely blood POCTs. We used a comprehensive search strategy, and by synthesising qualitative rather than quantitative studies we were able to gain an in-depth understanding of clinicians’ perspectives.

We limited our review to blood POCTs in primary care, high income country settings. Future research could compare attitudes amongst countries which have different primary care health systems; also the attitudes of other frontline clinicians such as paramedics or emergency department staff. We focused on blood tests; further research is needed to confirm whether the issues raised here apply to other types of POCTs (for example urine tests, respiratory samples).

Thematic synthesis is dependent on the quality of included studies, the themes and participant quotes which authors of these studies choose to present, and the interpretations of the reviewers. One of the included studies was poorly described and it was not possible to assess its rigour. Consequently we were cautious in interpreting its findings. Additionally it was relatively old (published 10 years before the next oldest study), and the POCTs used blood obtained by venipuncture rather than finger-prick blood samples, meaning that the findings might be out of date for current POCT practices. We minimised bias in selectivity and interpretation of synthesis results by having two authors independently extract and explore themes in the data, and discussing themes between all authors to reach consensus. We found broad agreement between our descriptive themes and the conclusions of individual studies. None of us were involved in the included studies in any way. The main reviewers (CJ, JH) are not clinicians and did not have experience or preconceived opinions regarding POCTs, which strengthened our approach. To counter the risk that participants’ quotes were interpreted differently from how they were intended, other authors who are primary care clinicians (MT, CH) verified the themes and interpretations. All of the authors are involved in identifying and evaluating emerging diagnostic technologies. We are interested in the potential for them to positively impact primary care, and it is possible that we hold underlying positive attitudes towards the implementation of POCTs. However, we were careful to identify negative as well as positive attitudes towards POCTs, and we have reported these thoroughly.

There is a risk of publication bias, in that studies demonstrating negative attitudes or impacts of POCTs may be less likely to be published. In three of the included studies, manufacturers loaned or provided equipment, [17,18,21] although the authors state that the manufacturers had no role in the study or preparing of the manuscript. Despite the risk of bias, we identified a balance of barriers and facilitators to the widespread use of POCTs.

## Conclusions

A multitude of POCTs are on the market and new ones are constantly being developed. These are of interest to primary healthcare clinicians who face growing pressures to perform more tests, more rapidly, reducing referrals and risk of diagnostic errors [23]. The findings of this review confirm that from the perspectives of primary care clinicians, likely benefits of introducing POCTs include increased diagnostic certainty, more efficient care, and fewer (re)consultations.

This review has also highlighted several clear barriers to the implementation of POCTs. If they are to be implemented more widely, these barriers must be addressed, some by primary care and others elsewhere. The accuracy of POCTs in primary care populations, and the way this is presented to clinicians, must be addressed by industry. Policy makers and clinicians should carefully consider the role and impact of POCTs in primary care; in particular, attention should be paid to impacts on GPs’ roles so that clinical expertise is enhanced rather than undermined. Furthermore, it is essential to define more clearly the different situations and patients in which POCTs are useful. In the context of reductions in health service funding, and importance of primary care commissioning, attention must be paid to how the costs of POCTs will be funded.

This review highlights that exploring the attitudes of primary care clinicians is integral to understanding if and how POCTs may become adopted more widely. It is possible that emphasizing the benefits and addressing the concerns highlighted in this review may lead to wider adoption of POCTs in primary care.

## Competing interests

The authors declare that they have no competing interests.

## Authors’ contributions

All authors conceptualized the study and participated in study design. NR conducted the literature search. CJ and JH determined eligibility of studies, and extracted and synthesized the data. MT checked eligibility of included studies. CJ drafted the manuscript. All authors read and approved the final manuscript.

## Pre-publication history

The pre-publication history for this paper can be accessed here:

http://www.biomedcentral.com/1471-2296/14/117/prepub

## Supplementary Material

Additional file 1 **Search strategy: Medline (OvidSP).** Primary care clinicians’ attitudes towards point of care testing. Click here for file
