# Supplementary material for: Primary care clinicians’ attitudes towards point-of-care blood testing: a systematic review of qualitative studies
Source: BMC Fam Pract. 2013 Aug 14;14:117. doi: 10.1186/1471-2296-14-117 (PMC3751354; doi:10.1186/1471-2296-14-117)
Supplement: Additional file 1 — Search strategy: Medline (OvidSP). Primary care clinicians’ attitudes towards point of care testing. [file 1471-2296-14-117-S1.docx]

***Additional file 1. Search strategy: Medline (OvidSP)***

| \| 1 \| ((immediate$ or rapid$ or same time or same visit or near patient or instant$ or portable or bedside or bed-side) adj3 (test$ or turnaround or analys$ or analyz$ or measure$ or assay$ or monitor*)).tw. \|  \| \| --- \| --- \| --- \| \| 2 \| (poc or poct or "point of care").tw. \|  \| \| 3 \| Point-of-Care Systems/ \|  \| \| 4 \| 3 or 2 or 1 \|  \| \| 5 \| general practice/ or family practice/ \|  \| \| 6 \| general practitioners/ or physicians, family/ or physicians, primary care/ \|  \| \| 7 \| Community Health Services/ \|  \| \| 8 \| Community Health Nursing/ \|  \| \| 9 \| Community Pharmacy Services/ \|  \| \| 10 \| Ambulatory Care/ \|  \| \| 11 \| Primary Health Care/ \|  \| \| 12 \| Office Visits/ \|  \| \| 13 \| ((general or family) adj2 practi*).ti,ab. \|  \| \| 14 \| ((general or family) adj2 physician*).ti,ab. \|  \| \| 15 \| (primary adj2 (care or healthcare)).ti,ab. \|  \| \| 16 \| (community adj2 (care or healthcare or service* or nurs* or clinic?)).ti,ab. \|  \| \| 17 \| (ambulatory adj2 (care or healthcare or service* or clinic?)).ti,ab. \|  \| \| 18 \| "out of hours".ti,ab. \|  \| \| 19 \| 5 or 6 or 7 or 8 or 9 or 10 or 11 or 12 or 13 or 14 or 15 or 16 or 17 or 18 \|  \| \| 20 \| 4 and 19 \|  \| \| 21 \| interview*.mp. \|  \| \| 22 \| px.fs. \|  \| \| 23 \| qualitative.tw. \|  \| \| 24 \| Qualitative Research/ \|  \| \| 25 \| 21 or 22 or 23 or 24 \|  \| \| 26 \| 20 and 25 \|  \| \| 27 \| (Qualitative systematic review* or (systematic review and qualitative)).mp. [mp=title, abstract, original title, name of substance word, subject heading word, protocol supplementary concept, rare disease supplementary concept, unique identifier] \|  \| \| 28 \| (evidence synthesis or realist synthesis).mp. [mp=title, abstract, original title, name of substance word, subject heading word, protocol supplementary concept, rare disease supplementary concept, unique identifier] \|  \| \| 29 \| (Qualitative and synthesis).mp. [mp=title, abstract, original title, name of substance word, subject heading word, protocol supplementary concept, rare disease supplementary concept, unique identifier] \|  \| \| 30 \| (meta-synthesis* or meta synthesis* or metasynthesis).mp. [mp=title, abstract, original title, name of substance word, subject heading word, protocol supplementary concept, rare disease supplementary concept, unique identifier] \|  \| \| 31 \| (meta-ethnograph* or metaethnograph* or meta ethnograph*).mp. [mp=title, abstract, original title, name of substance word, subject heading word, protocol supplementary concept, rare disease supplementary concept, unique identifier] \|  \| \| 32 \| (meta-study or metastudy or meta study).mp. [mp=title, abstract, original title, name of substance word, subject heading word, protocol supplementary concept, rare disease supplementary concept, unique identifier] \|  \| \| 33 \| 27 or 28 or 29 or 30 or 31 or 32 \|  \| \| 34 \| 4 and 19 and 33 \|  \| \|  \|  \|  \| |
| --- | --- | --- | --- | --- | --- | --- | --- | --- | --- | --- | --- | --- | --- | --- | --- | --- | --- | --- | --- | --- | --- | --- | --- | --- | --- | --- | --- | --- | --- | --- | --- | --- | --- | --- | --- | --- | --- | --- | --- | --- | --- | --- | --- | --- | --- | --- | --- | --- | --- | --- | --- | --- | --- | --- | --- | --- | --- | --- | --- | --- | --- | --- | --- | --- | --- | --- | --- | --- | --- | --- | --- | --- | --- | --- | --- | --- | --- | --- | --- | --- | --- | --- | --- | --- | --- | --- | --- | --- | --- | --- | --- | --- | --- | --- | --- | --- | --- | --- | --- | --- | --- | --- | --- | --- | --- |
